# Inhibitory Effects of Antimicrobial Photodynamic Therapy with Curcumin on Biofilm-Associated Gene Expression Profile of *Aggregatibacter actinomycetemcomitans*

**Published:** 2018-05

**Authors:** Maryam Pourhajibagher, Nasim Chiniforush, Abbas Monzavi, Hamidreza Barikani, Mohammad Moein Monzavi, Shaghayegh Sobhani, Sima Shahabi, Abbas Bahador

**Affiliations:** 1 Researcher, Dental Research Center, Dentistry Research Institute, Tehran University of Medical Sciences, Tehran, Iran; 2 Researcher, Laser Research Center of Dentistry, Dentistry Research Institute, Tehran University of Medical Sciences, Tehran, Iran; 3 Professor, Laser Research Center of Dentistry, Dentistry Research Institute, Tehran University of Medical Sciences, Tehran, Iran; Department of Prosthodontics, School of Dentistry, Tehran University of Medical Sciences, Tehran, Iran; 4 Researcher, Dental Implant Research Center, Dentistry Research Institute, Tehran University of Medical Sciences, Tehran, Iran; 5 Dentist, Private Practice, Tehran, Iran; 6 Professor, Laser Research Center of Dentistry, Dentistry Research Institute, Tehran University of Medical Sciences, Tehran, Iran; Department of Dental Biomaterials, School of Dentistry, Tehran University of Medical Sciences, Tehran, Iran; 7 Associate Professor, Department of Microbiology, School of Medicine, Tehran University of Medical Sciences, Tehran, Iran

**Keywords:** *Aggregatibacter actinomycetemcomitans*, Biofilms, Curcumin, Periodontitis, Quantitative Real-Time Polymerase Chain Reaction

## Abstract

**Objectives:**

Periodontitis is an inflammation of periodontal tissues that is caused by the biofilm of periodontal pathogens. *Aggregatibacter actinomycetemcomitans* (*A. actinomycetemcomitans*) is an opportunistic periodontopathogen that can be the cause of periodontal diseases via fimbriae as a virulence factor. In this study, we aimed to determine the expression level of *A. actinomycetemcomitans rcpA* gene as a virulence factor associated with biofilm formation after antimicrobial photodynamic therapy (aPDT) as a relatively new therapeutic modality.

**Materials and Methods:**

To determine sub-lethal doses of aPDT against *A. actinomycetemcomitans* ATCC 33384 strain, we used curcumin (CUR) as a photosensitizer at a final concentration of 40 μmol/ml, which was excited with a light-emitting diode (LED) at the wavelength of 450 nm. Quantitative real-time polymerase chain reaction (qRT-PCR) was then applied to monitor *rcpA* gene expression in *A. actinomycetemcomitans*.

**Results:**

10–40 μmol/ml of CUR caused a significant reduction in the growth of *A. actinomycetemcomitans* compared to control group (P<0.05). Also, the cell viability of *A. actinomycetemcomitans* was significantly decreased after more than four minutes of LED irradiation. Therefore, the sub-lethal dose of aPDT against *A. actinomycetemcomitans* was 5 μmol/ml of CUR with three minutes of LED irradiation at a fluency of 180–240 J/cm^2^, which reduced the expression of the *rcpA* gene by approximately 8.5-fold.

**Conclusions:**

aPDT with CUR leads to decreased cell survival and virulence of *A. actinomycetemcomitans*. Thus, CUR-aPDT can be used as an alternative approach for the successful treatment of periodontitis *in vivo*.

## INTRODUCTION

Periodontitis is an infection-induced inflammatory disease caused by multispecies biofilm models of periodontal pathogens [1]. Biofilms are surface-attached microbial complex communities that are embedded in a matrix of extracellular polymeric substances [2]. Microbial biofilms are resistant to antimicrobial agents and can enhance the survival of the microbiota [3]. *Aggregatibacter actinomycetemcomitans* (*A. actinomycetemcomitans*), an opportunistic periodontopathogen with various virulence factors, is able to resist the clearance attempts because of its protective extracellular matrix and due to the presence of resistant cells [4]. The tight-adherence (tad) gene locus in *A. actinomycetemcomitans* is necessary for surface attachment and biofilm formation. Different proteins encoded by the tad locus form *A. actinomycetemcomitans* fimbriae. Rough colony protein A (RcpA), a multimeric complex in the outer membrane of *A. actinomycetemcomitans*, forms a secreting channel that secretes the fimbriae components to outside the outer membrane [5]. The *rcpA* is a virulence factor that is known to be particularly important in *A. actinomycetemcomitans* isolates to form tight biofilms. It allows for interactions with host epithelial cells, which can lead to an up-regulation of biofilm-associated bacterial genes [6]. Several studies have suggested that antimicrobial photodynamic therapy (aPDT), also known as photochemotherapy and photoactivated disinfection [7], is a safe alternative strategy for periodontal therapy and can significantly change the cell survival of *A. actinomycetemcomitans* [8]. During aPDT, a photosensitizing chemical substance and a specific wavelength of light, used in conjunction with molecular oxygen, elicit cell death in microorganisms [7]. Recently, curcumin (CUR), a new class of photosensitizers, has been introduced for aPDT. CUR [1E,6E-1,7-bis(4-hydroxy-3-methoxyphenyl)-1,6-heptadiene-3,5-dione] is a yellow dye (also known as the spice turmeric) isolated from *Curcuma longa*. Recent investigations have shown that CUR is a potent photoactivatable substance that exhibits a variety of pharmacological properties such as antimicrobial, anti-inflammatory, anticancer, and antitumor activities [9,10].

To date, no data are available on the effects of aPDT with CUR, as a photosensitizer, on the expression of *A. actinomycetemcomitans* virulence factors. In the current study, we report the results of the effects of CUR-mediated aPDT on the expression of *rcpA* gene in *A. actinomycetemcomitans* cells surviving the PDT.

## MATERIALS AND METHODS

### Bacterial strain and growth conditions:

*A. actinomycetemcomitans* ATCC 33384 strain, purchased from Institute of Microbiology, ETH Zurich, Switzerland, was cultured in microaerophilic conditions (<20% O_2_) for 48 hours at 37°C in a culture medium that was prepared by using brain heart infusion (BHI) agar (Merck KGaA, Darmstadt, Germany) to which the following compounds were added: 5% defibrinated sheep blood (Sigma-Aldrich Co., Ltd., Dorset, United Kingdom), 5 g/L of yeast extract (Merck KGaA, Darmstadt, Germany), 5 mg/L of hemin (Sigma-Aldrich Co., Ltd., Dorset, United Kingdom), and 1 mg/L of menadione (Sigma-Aldrich Co., Ltd., Dorset, United Kingdom). The strain was then inoculated into freshly prepared tubes containing BHI broth (Merck KGaA, Darmstadt, Germany), and the cell density was adjusted to 1.5×10^8^ cells/ml as verified by a spectrophotometer (Eppendorf BioPhotometer, Hamburg, Germany) to measure the optical density at 600 nm (OD600) and to count the colonies.

### Photosensitizing agent and light source:

CUR (Merck KGaA, Darmstadt, Germany), a photosensitizer, was prepared in 0.05% dimethyl sulfoxide (DMSO) at a final concentration of 40 μmol/ml. A light-emitting diode (LED, DY400-4, Denjoy Dental Co., Ltd., Shenzhen, China) at the wavelength of 450 nm with an output intensity of 1000–1400 mW/cm^2^ was used as a light source. The output powers were measured by a power meter (LaserPoint s.r.l, Milano, Italy) during the experiment.

### Determination of sub-lethal doses of CUR, light source, and aPDT:

The sub-minimum inhibitory concentration (sMIC) of CUR against *A. actinomycetemcomitans*, which is defined as a sub-lethal dose, was determined based on a previous study [11]. Briefly, 100 μl of 2X BHI broth was added to the wells of a round-bottom 96-well microplate (TPP AG, Trasadingen, Switzerland), and 100 μl of CUR was poured into the wells of the first column and was diluted two-fold stepwise. Afterward, 100 μl of *A. actinomycetemcomitans* suspension with 1.5×10^6^ colony-forming units (CFUs)/ml was added to each well. In this study, the wells containing only *A. actinomycetemcomitans* suspension and the wells containing BHI broth without *A. actinomycetemcomitans* suspension or CUR were used as positive and negative controls, respectively. The microplate was incubated in the dark for 5 minutes at room temperature (25±2°C) in microaerophilic conditions. Next, 10 μl from the contents of each well was cultured in an enriched BHI agar plate which its components were described above.

The plates were incubated for 48 hours at 37°C in microaerophilic conditions. FUs/ml were determined based on the Miles and Misra method [12]. The sub-lethal dose of LED irradiation time against *A. actinomycetemcomitans* was determined according to a previous study [11]. In this method, 300 μl of the free-floating *A. actinomycetemcomitans* in the planktonic suspension at the final concentration of 1.5×10^5^ CFU/ml was placed inside the microplate’s wells.

**Fig. 1:**
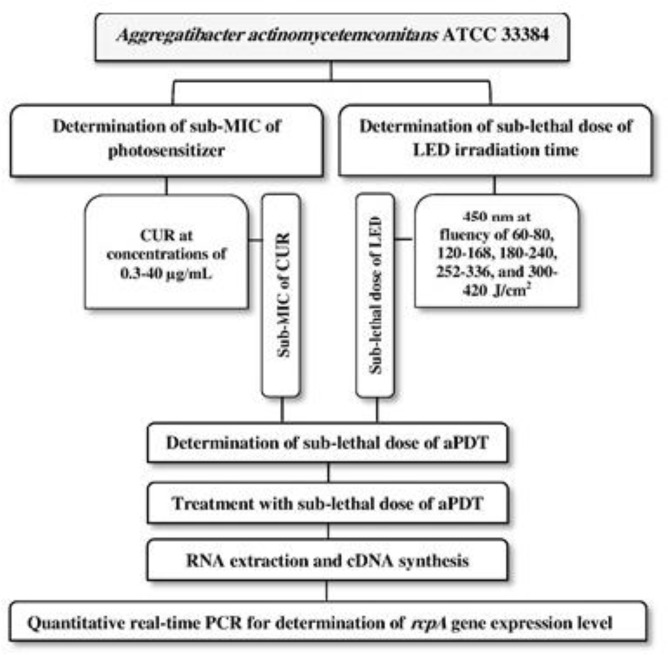
Flowchart of the experimental steps

The LED in continuous mode was applied with an output power of 1000–1400 mW/cm^2^ for 1, 2, 3, 4, and 5 minutes with energy densities of 60-80, 120–168, 180–240, 252–336, and 300–420 J/cm^2^, respectively. The LED probe was fixed at a 1-mm distance above each well’s surface. A black paper was placed under the microplate to prevent beam reflection from the tabletop during LED irradiation. Calculations of *A. actinomycetemcomitans* CFUs/ml were performed according to the method mentioned above. Eventually, sub-lethal doses of aPDT were specified based on a previous study [13]. In brief, 100 μl of 2X BHI broth was added to the wells of the round-bottom 96-well microplate, and 100 μl of 2X MIC CUR was then added and serially diluted two-fold to 1.8 MIC. Finally, 100 μl of *A. actinomycetemcomitans* suspension with the concentration of 1.5×10^6^ CFU/ml was poured into each well. The microplate was incubated for 5 minutes in the dark at room temperature in microaerophilic conditions before LED irradiation. The treated *A. actinomycetemcomitans* suspension in the well was exposed to the sub-lethal dose of LED irradiation time. Sub-lethal doses of aPDT were determined according to the methods mentioned previously [13]. The flowchart of the experimental steps is shown in Figure 1.

### Sub-lethal doses of aPDT against A. actinomycetemcomitans isolates:

After determining the sub-lethal doses of aPDT in the previous section, the *A. actinomycetemcomitans* isolate was exposed to the sub-lethal dose of CUR and to the sub-lethal dose of LED irradiation time. The untreated *A. actinomycetemcomitans* isolate which did not receive sub-lethal doses of aPDT was used as a control group.

### RNA preparation and complementary DNA (cDNA) synthesis:

The total RNA of treated and untreated *A. actinomycetemcomitans* isolates was extracted by using an RNA Extraction Kit (GeneAll Hybrid-RTM RNA purification; GeneAll Biotechnology Co., Ltd., Korea) in accordance with the manufacturer’s recommendations. NanoDrop® ND-1000 spectrophotometer (Thermo Fisher Scientific Inc., Waltham, MA, USA) at 260 nm and 280 nm (A260/A280 ratio) was used to evaluate the quality of the total extracted RNA.

The total extracted RNA was treated by RNase-free DNase I (Thermo Fisher Scientific GmbH, Bremen, Germany) to eliminate the genomic DNA from RNA that could remain after the extraction. First-strand cDNA was then synthesized through random hexamer primed reactions by using a RevertAid First Strand cDNA Synthesis Kit (Thermo Fisher Scientific Inc., Waltham, MA, USA) according to the manufacturer’s protocol.

### Quantitative real-time polymerase chain reaction (qRT-PCR) and gene expression analysis:

The primers for *A. actinomycetemcomitans* were determined based on *rcpA* and comparative 16S ribosomal RNA (*16S rRNA*) gene sequences by utilizing Primer3 Input version 0.4.0 online software (http://frodo.wi.mit.edu/primer3/).

Each nucleotide sequence was further evaluated by using the nucleotide algorithm of the Basic Local Alignment Search Tool (BLAST; https://blast.ncbi.nlm.nih.gov/). Also, the primer specificity was determined by melting curve analysis. The qRT-PCR assays were performed in triplicate by the Line-GeneK Real-Time PCR Detection System (Bioer Technology Co., Ltd., Hangzhou, China). qRT-PCR process contained the following reaction components in a 20-μl reaction: 1 μl of cDNA (corresponding to the cDNA transcribed from approximately 10 ng of RNA), 10 μl of SYBR Premix Ex Taq II (2x) (Takara Bio Inc., Otsu, Japan), 1 μl of forward and 1μl of reverse primer, which are shown in Table 1, and 7 μl of sterile distilled water. The thermal cycling conditions for qRT-PCR (25-μl reaction volume) included an initial denaturation of 5 minutes at 95°C, followed by 35 cycles at 95°C for 10 seconds, at 59°C for 15 seconds, and at 72°C for 15 seconds.

**Table 1.** Primer sequences used in the present study

**Gene**	**Primer**	**Sequences (5′– 3′)^a^**	**Tm (°C)**	**Amplicon Size (bp)**
*rcpA*	F	TGGGCATTAACTGGAGCCAC	60	72
	R	ATCCACCTCCGAAACCGAAG		
*16S rRNA*	F	AAGCACCGGCTAACTCCGT	60	63
	R	TTCCGATTAACGCTCGCAC		

F=Forward primer, R=Reverse primer, Tm=Melting temperature, bp=Base pair

a Nucleotides

### Statistical analysis:

One-way analysis of variance (ANOVA) and Tukey’s post-hoc tests were performed to statistically analyze the data. The changes in the expression level of target gene were analyzed by using the method adopted by Livak and Schmittgen [14]. All the experiments were done in triplicate, and P-values lower than 0.05 were considered statistically significant.

## RESULTS

**Fig. 2:**
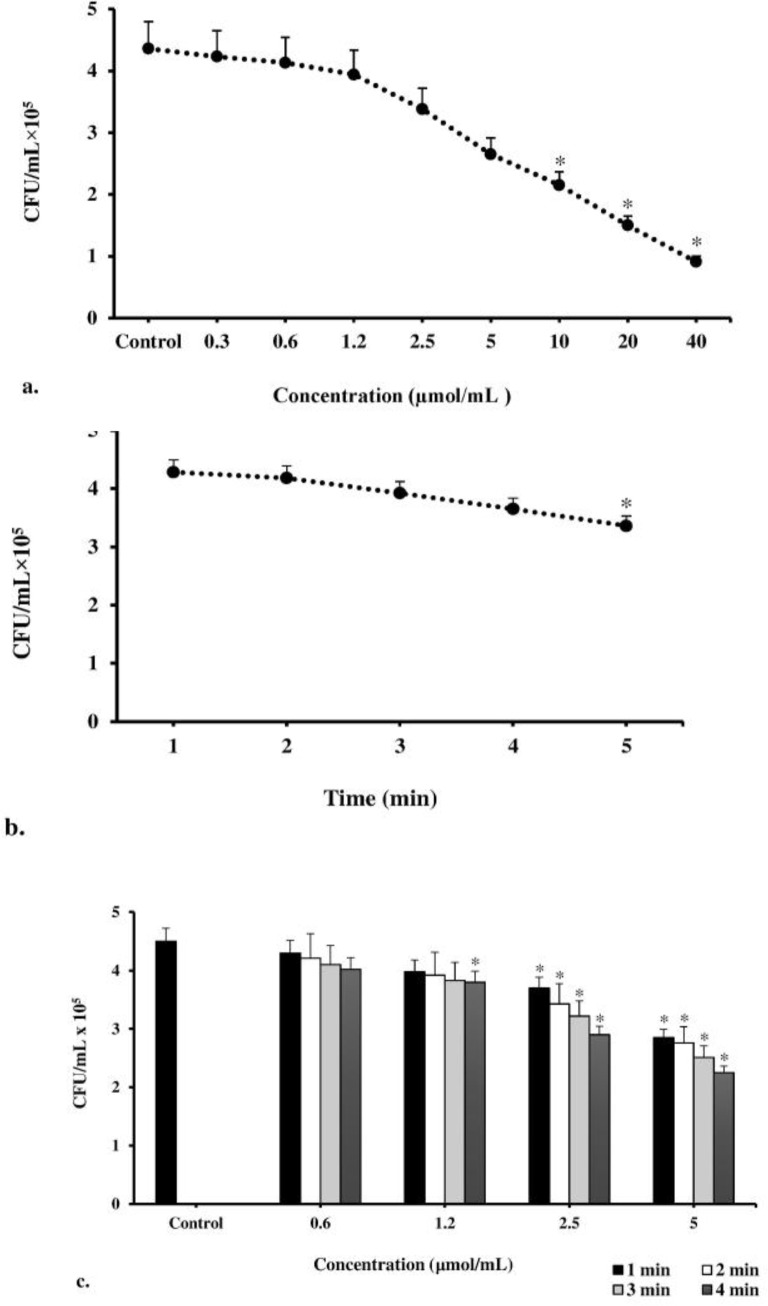
Minimum inhibitory concentration (MIC) of (a) Curcumin (CUR), (b) light-emitting diode (LED), and (c) antimicrobial photodynamic therapy (aPDT) against *Aggregatibacter actinomycetemcomitans*. *Significantly different from the control (P<0.05)

**Fig. 3:**
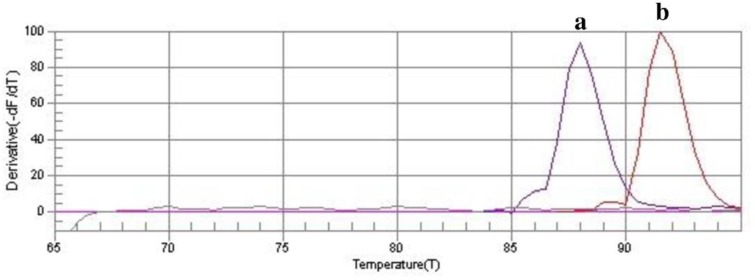
The melting curve profiles generated by real-time amplification to assess potential primer-dimer artifacts (or non-specific PCR product). a.*16S rRNA*, ***b.rcpA***

**Fig. 4:**
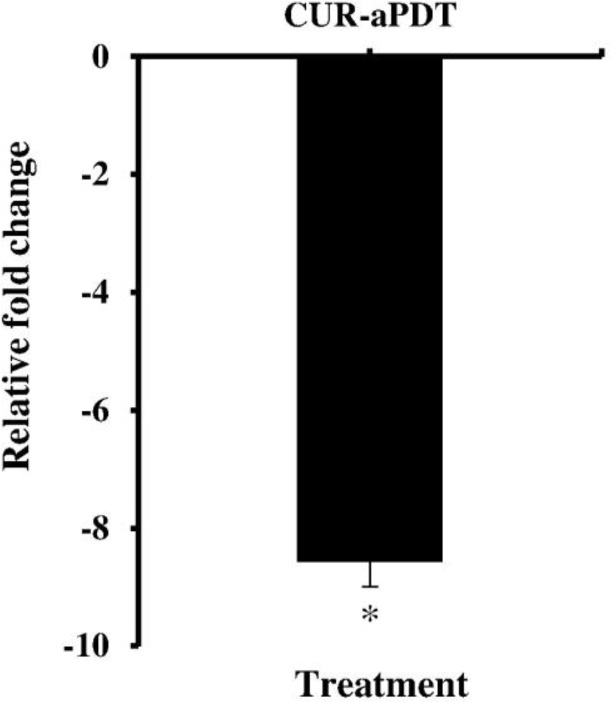
Effect of the sub-lethal dose of antimicrobial photodynamic therapy (aPDT) on the expression ratio of *rcpA* gene in *Aggregatibacter actinomycetemcomitans*. *Significantly different from the control (P<0.05)

According to the results of our study, CUR at 10–40 μmol/ml concentrations significantly reduced the cell survival of *A. actinomycetemcomitans* isolates from 50.5% to 79.1% compared to control group (untreated bacteria; P<0.05, Fig. 2a), whereas there was no statistically significant reduction when the concentration of CUR was increased from 0.3 to 5 μmol/ml. Therefore, the MIC and maximal sub-MIC doses of CUR against *A. actinomycetemcomitans* were found to be 5 and 2.5 μmol/ml, respectively. According to Figure 2b, our assays revealed that the cell viability of *A. actinomycetemcomitans* isolates was significantly reduced (30.8%) after 5 minutes of LED exposure with 300–420 J/cm^2^ energy density. There was no decrease in the count of *A. actinomycetemcomitans* cells in the other LED radiation times (P>0.05). Therefore, the sub-lethal dose of LED irradiation time was four minutes with an output light energy of 252–336 J/cm^2^. According to the results presented in Figure 2c, the maximal CUR-mediated sub-lethal dose of aPDT which exhibited a non-significant reduction in *A. actinomycetemcomitans* cell count was 1.2 μmol/ml of CUR plus three minutes of irradiation time at a fluency of 180–240 J/cm^2^. The results of each primer’s melting curve in Figure 3 show that the presence of a single curve for each primer implies the formation of a single product, which shows the specificity of the primers for target genes. As shown in Figure 4, maximal sub-lethal doses of aPDT presented a significant 8.5-fold reduction of rcpA in *A. actinomycetemcomitans* cells and can be effective in reducing the biofilm formation of *A. actinomycetemcomitans* isolates.

## DISCUSSION

Periodontal infection is a polymicrobial infection where hundreds of different microorganism species are present in a periodontal lesion [15]. *A. actinomycetemcomitans* is an opportunistic periodontopathogen that can firmly attach to dental surfaces and subgingival crevicular epithelial cells to form subgingival biofilms [16,17].

*A. actinomycetemcomitans* demonstrates a great genetic diversity in its ability as a periodontopathogen to express different virulence factors [18]. Fimbriae are important in the initial adhesion of *A. actinomycetemcomitans* to dental surfaces and in biofilm formation. It has been reported that the tad locus contains 14 genes, including *rcpA*, that are found tandemly located downstream of the fimbriae. Overall, *rcpA* appears to play a critical role in the colonization of *A. actinomycetemcomitans* and in the formation of biofilms [5].

Interestingly, the microbiota in the biofilm phase are up to 1000-fold more tolerant and/or resistant to antimicrobial agents compared to the planktonic phase [19]. Also, the transformation efficiency rates can be 10- to 600-fold higher in biofilms than in planktonic cells [20].

*A. actinomycetemcomitans* can create persistent infections in periodontal regions and can promote resistance to antimicrobial agents and periodontal treatment via biofilm formation [19]. Therefore, the development of a new antimicrobial approach with fewer complications is necessary when there is a possibility of acquired resistance to antimicrobial elements by *A. actinomycetemcomitans.*

Previous studies have shown that aPDT, as a noninvasive therapeutic modality, is a new promising strategy to eliminate pathogenic microbiota [21–23]. During aPDT, a pharmacologically inert chromophore called a photosensitizer is used with a low-power laser of an appropriate wavelength. The photosensitizer is stimulated through exposure to light. In the presence of oxygen, the excited photosensitizer can release reactive nitrogen species (RNS) and oxygen species (ROS), such as superoxide, hydroxyl radicals, and hydrogen peroxide, which are harmful to cell membrane integrity and can cause biological death [21].

The antibacterial and antibiofilm effects of aPDT with different photosensitizers have been previously assessed. Haag et al [24] evaluated the in-vitro antimicrobial efficacy of aPDT against periodontopathogenic bacteria and reported that aPDT with methylene blue (MB) resulted in a significant reduction of the surviving bacteria. The results of their study showed that the range of log10-reduction was about 39% to 100% with MB-aPDT against *A. actinomycetemcomitans* [24]. On interpreting the results of previous studies, it becomes obvious that aPDT could reduce the load of periodontopathogenic bacteria in cases of chronic periodontitis, aggressive periodontitis, and peri-implantitis [25].

On the other hand, Eick et al [26] found that toluidine blue O (TBO) and a diode laser (625–635 nm) were effective in reducing the viability of *A. actinomycetemcomitans*.

CUR is a hydrophobic photosensitizer that is soluble in DMSO, acetone, ethanol, and oils, and it has an absorption spectrum in the ultraviolet UV)/blue wavelength range of 300–500 nm with a maximum absorption at 430 nm [27,28].

Najafi et al [29] conducted a study to investigate the effect of aPDT with CUR against *A. actinomycetemcomitans* and demonstrated that aPDT with 5 mg/ml of CUR decreased the *A. actinomycetemcomitans* CFUs/ml by approximately 65% in comparison with control group. Araújo et al [30] reported the photodynamic effects of CUR against cariogenic pathogens. A reduction of up to 99.99% in the viability of *Streptococcus mutans* was observed when 1.5 g/L of CUR was used, whereas the reduction of the cell viability of *Lactobacillus acidophilus* was considerably lower (37.6%) after CUR-aPDT [30].

The results of the present study revealed that CUR at the concentrations above 5 μmol/ml can significantly reduce the *A. actinomycetemcomitans* CFUs/ml. To the best of our knowledge, this is the first experiment that has determined the effects of aPDT with CUR on the expression level of *rcpA* gene in *A. actinomycetemcomitans*. Other studies have shown that aPDT can significantly reduce the pathogenicity of other microorganisms [31,32]. Based on the results obtained from the current experiment, *A. actinomycetemcomitans rcpA* gene was down-regulated 8.5-fold, which can reduce the *A. actinomycetemcomitans* strain’s ability to form biofilms.

One of the limitations of this study was in-vitro analysis without in-vivo assays. Further studies should be conducted on CUR-aPDT. Also, in-vivo evaluations are needed to confirm the clinical relevance of these results. In addition, CUR needs to be further improved for clinical use.

## CONCLUSION

The results of the present study indicate that CUR-mediated aPDT shows antibacterial potential against *A. actinomycetemcomitans* and can repress the expression level of *rcpA* which is a gene involved in biofilm formation. It can be concluded that CUR-aPDT is a useful alternative strategy for periodontal therapy.
